# Change in hip laxity after anterior capsular suture in total hip arthroplasty using direct anterior approach

**DOI:** 10.1038/s41598-024-52636-w

**Published:** 2024-01-27

**Authors:** Takashi Imagama, Yuta Matsuki, Tomoya Okazaki, Takehiro Kaneoka, Takehiro Kawakami, Kazuhiro Yamazaki, Takashi Sakai

**Affiliations:** Department of Orthopaedic Surgery, Yamaguchi University Graduate School of Medicine, 1-1-1, Minamikogushi, Ube, 7558505 Japan

**Keywords:** Osteoarthritis, Rheumatoid arthritis, Musculoskeletal abnormalities, Surgery, Reconstruction

## Abstract

It is clinically unclear whether anterior capsular suture improves hip laxity in total hip arthroplasty using direct anterior approach (DAA-THA). This study aimed to clarify the impact of anterior capsular suture for hip laxity in DAA-THA. In this study, 121 hips of 112 patients who underwent DAA-THA were prospectively enrolled. Mean age was 64.7 ± 10.1 years, and the subjects consisted of 35 hips in 32 men and 86 hips in 80 women. To evaluate hip laxity after implantation, axial head transfer distance (HTD) when the hip was pulled axially at 15 kg was compared before and after anterior capsular suture at the hip intermediate and 10° extension positions. HTD in the intermediate and 10° extension positions averaged 5.9 ± 4.6 mm and 6.3 ± 4.6 mm before the suture, and 2.6 ± 2.7 mm and 2.9 ± 3.1 mm after the suture, respectively. HTD after the suture significantly decreased in both hip positions (*p* < 0.0001). The amount of change by the suture was greater in cases with greater pre-suturing HTD. In DAA-THA, the anterior capsular suture significantly improved hip laxity against axial traction force, it may contribute to improvement of postoperative hip stability, especially in cases with greater laxity before the suture.

## Introduction

Total hip arthroplasty (THA) has been reported to yield good long-term results in patients with hip pain and gait disturbances caused by hip osteoarthritis or other deformities^1^. However, dislocation is one of the most important postoperative complications^2^. Although the frequency of initial dislocation after THA has been reported to be 0.4%-3.9%^2–4^, it has also been reported that 16% of the initial dislocations shift to recurrent dislocations^5^. Laxity of soft tissues around the hip has been described as one of factors of dislocation after THA^6^.

Direct anterior approach (DAA) for THA was reported to reduce the postoperative dislocation rate compared to posterior approach^3^, because DAA does not require muscle–tendon dissection in most cases. However, the anterior capsule must be incised in THA using DAA (DAA-THA). The hip capsule contains the iliofemoral, pubofemoral, and ischiofemoral ligaments. The iliofemoral ligament, located in the anterior capsule, is divided into vertical and horizontal bands. The vertical band controls external rotation in hip extension position and the horizontal band controls internal or external rotation in hip extension position and external rotation in flexion position^7,8^. The hip capsule is considered to contribute to the hip stability, and it is conceivable that the incision of anterior capsule may play a role in hip stability in DAA-THA. However, there is no consensus on how to evaluate soft tissue tension in THA. Khair et al. reported that the more anterior capsule of native hip was incised, the more joint laxity was increased against axial traction force in a cadaver study^9^. And it has been reported that hip laxity against axial traction force affected recurrent dislocation after THA. Ogawa et al. described that patients with recurrent dislocation following THA had lower soft tissue tension against distal traction force than no dislocation cases^10^. From these perspectives, we believe that soft tissue stabilization against axial traction force is important in THA. To our knowledge, there have been no reports on whether anterior hip capsular suture improves hip laxity against axial traction force in DAA-THA.

In this study, we hypothesized that anterior capsular suture would significantly contribute to improvement of hip laxity in DAA-THA, and the effect is higher in cases with greater laxity before the suture. Thus, we aimed to reveal the changes in hip laxity against axial traction force before and after anterior capsular suture intraoperatively in DAA-THA. Additionally, to determine in which cases the suture is more effective, we evaluated correlation between the transfer distance of the head before suture and the distance reduced by the suture. Moreover, we examined the relationship between leg lengthening or offset changes and the transfer distance before the suture, because these changes in THA affect soft tissue tension around the hip.

## Methods

### Patients

Between June 2020 and April 2022, 131 hips in 122 consecutive patients who received primary DAA-THA with anterior capsular suture at our institute were prospectively enrolled in this study. Patients whose intraoperative hip laxity could not be evaluated were excluded (lack of data [n = 3], fluoroscopy trouble [n = 2], anterior capsules were detached intraoperatively [n = 3]). Two rheumatoid arthritis patients were also excluded due to possible weakness of the joint capsule. Finally, 121 hips in 112 patients were included in the study. The estimated sample size was calculated with G power 3.1 (Heinrich Heine University, Düsseldorf, Germany), and the required number of cases was 35 with effect size of 0.5, α = 0.05, and power of 0.8.

The mean age of patients was 64.7 ± 10.1 years; there were 35 hips in 32 men and 86 hips in 80 women. Mean body mass index was 23.5 ± 3.2 kg/m^2^. Underlying diseases included: osteoarthritis of the hip in 99 hips in 92 patients; osteonecrosis of the femoral head in 17 hips in 15 patients; and rapidly destructive coxarthropathy in 5 hips in 5 patients. The Crowe classification was used to assess the degree of upward displacement of the femoral head for dysplasia^11^. The number of hips with type I, II, III, and IV was 72, 21, 6, and 0, respectively. The implants were PINNACLE acetabular cup (DepuySynthes, Warsaw, IN, USA) in 94 hips; Trident hemispherical shell (Stryker, Kalamazoo, MI, USA) in 27 hips; Corail stem (DepuySynthes, Warsaw, IN, USA) in 101 hips; Accolade II (Stryker, Kalamazoo, MI, USA) in 18 hips; and GS Taper stem (Teijin Nakashima Medical, Okayama, Japan) in 2 hips. In all cases ceramic heads and flat polyethylene liners were used. The head sizes were 36 mm, 32 mm, and 28 mm in 36, 78, and 7 hips, respectively. The surgeons basically chose PINNACLE acetabular cup and Corail stem, and other implants were used at the surgeon's discretion depending on preoperative planning.

### Surgical procedure

**Figure 1:**
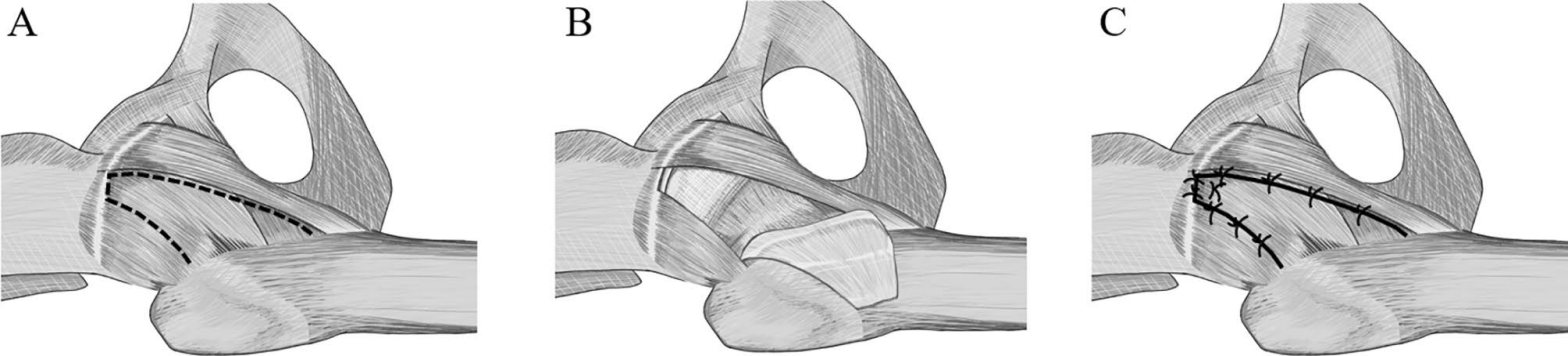
(**A**) The anterior capsule was incised in a triangular shape (dashed line) to preserve the base of the femoral side of the capsule (right hip shown). (**B**) The incised flap-shaped anterior capsule was inverted to peripheral side, and manipulation was performed into the joint. (**C**) After implant insertion, the flap-shaped anterior capsule was returned, and the proximal remnant and the flap were tightly sutured. Then, 3 or 4 sutures were placed on the medial side, and the lateral side was sutured with 2 or 3 stiches.

All patients underwent the same surgical procedure conducted by two senior surgeons with a normal operating table. Both surgeons had conducted DAA-THA in more than 100 cases before this study. All surgeries were performed under a combination of general and epidural anesthesia. The patient was placed in the supine position, and a longitudinal skin incision of approximately 8–10 cm was made peripherally from approximately 2 cm distally and 2 cm laterally from the anterior superior iliac spine. After exposing the anterior capsule, a triangular flap based on the femoral attachment was created (Fig. 1A); the capsular incision was made to preserve the vertical band of the iliofemoral ligament. The flap was inverted to the peripheral side to perform intra-articular manipulation (Fig. 1B). The cup was normally placed in the original position with an inclination of 40° and an anteversion of 15° based on the functional pelvic plane using fluoroscopy or CT-based navigation system (Stryker, Kalamazoo, MI, USA). Subsequently, superolateral release was performed to elevate the femur and insert the stem, while the short external rotators of the hip were typically preserved. The stem was inserted according to the preoperative plan, allowing the anteversion angle of the stem to go from 20° to 30°, and the leg length and global offset were adjusted to the opposite hip as close as possible. After implantation and before suturing anterior capsule, it was checked in all cases that there was no anterior dislocation at 20° of extension and maximum external rotation of the hip. Subsequently, capsular suture was performed at the proximal side with modified Kessler suture and interrupted suture using No. 5 Ethibond Excel (Ethicon, Bridgewater, NJ, USA), and interrupted suture was performed in the remaining parts (Fig. 1C). In 3 of the 6 cases, it was difficult to suture the anterior capsule to the original position. In these cases, the femoral anterior capsular attachment was partially dissected to increase the mobility of capsule and the anterior capsule sutured to the soft tissue near the pelvic capsular attachment and the vertical band of iliofemoral ligament.

### Method of hip laxity assessment against axial traction

**Figure 2:**
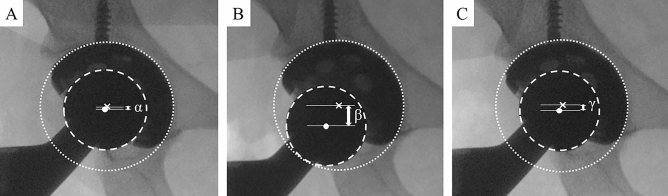
The dotted line is the circle along the cup. The dashed line is the circle along the head. The X mark and white dot represent the center of the cup and head, respectively. The distance between the lines parallel to the bilateral inter-teardrop line passing through these marks was defined as center-head distance (CHD: arrow at both ends). CHD without traction before the capsular suture is α (**A**), CHD with traction before the capsular suture is β (**B**), and CHD with traction after the capsular suture is γ (**C**). Head transfer distance (HTD) before the suture = β – α, HTD after the suture = γ – α, distance controlled by the suture (DCS) = HTD before the suture – HTD after the suture.

**Figure 3:**
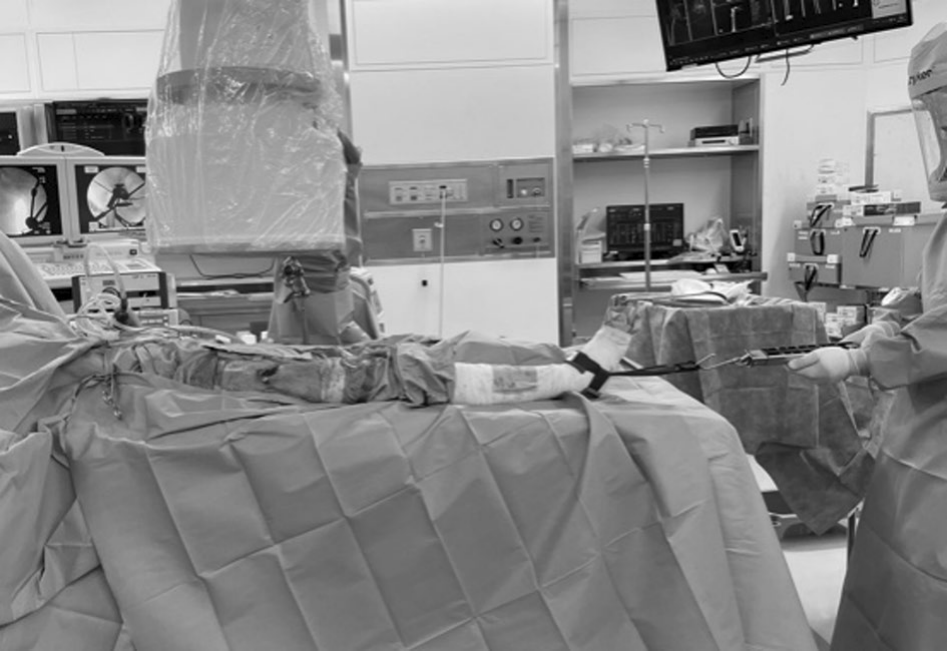
An ankle band was placed to the patient's ankle joint, and traction was applied at 15 kg to the axial direction along the lower limb using spring scale. Antero-posterior views of the hip with and without traction were taken by fluoroscopy in the hip intermediate and 10° extension positions before and after the suture.

After implantation and before suturing anterior capsule, the ankle band (Smith & Nephew, Watford, UK) which is used for ankle arthroscopy was attached to the ankle joint with the operating table completely flat, and the position of the artificial head before traction was recorded in the antero-posterior view of the hip under fluoroscopy (Fig. 2A). Subsequently, a spring scale (Shinwa Rules Co. Ltd., Niigata, Japan) was used to pull 15 kg to the distal direction (Fig. 3), and the images were recorded likewise (Fig. 2B). This manipulation was performed at the intermediate and 10° extension positions of the hip with neutral rotation and 0° of abduction twice each. The reason for evaluating at extension position is the risk position of anterior dislocation in cases of anterior capsular dissection^12^. After anterior capsular suture the images were recorded using the same method (Fig. 2C). Regarding traction force, in a cadaver study, the average for opening the joint space by 6 mm was 183 N when the anterior capsule of the native hip was incised by 4 cm^9^. Thus, the axial traction force was set to 15 kg in this study.

With respect to analysis, vertical distance between the center of the cup and head on the image was measured using the image analysis software, IC measure (The imaging source Co., Ltd., Taipei, Taiwan). The actual head size was used to calibrate the head size on the image. The value was defined as the cup-head center distance (CHD). In addition, the value obtained by subtracting CHD before traction from CHD during hip traction was defined as head transfer distance (HTD). CHD and HTD were expressed as the mean value calculated from two images of each. The value obtained by subtracting HTD after suturing anterior capsule from HTD before the suture was considered the distance controlled by the suture (DCS) (Fig. 2). These measurements were performed by two examiners who were not involved in the medical treatment, and average values ​​were used in this study. Intraclass correlation coefficient (ICC) was calculated on 30 randomly selected cases to confirm the reproducibility of the measurements. The measurement for intra-examiner error was conducted at least four weeks apart. ICCs for inter-examiner error and intra-examiner error were 0.994 and 0.996, respectively.

HTD before and after the suture were compared to determine the effect of the suture on hip laxity after DAA-THA.

### Measurement of leg length and global offset

Using the antero-posterior view of radiographic images, leg length was measured as the distance of lesser trochanter from the bilateral inter-teardrop line. The global offset was measured as the distance between the pubic symphysis and the long axis of the proximal femur. The values were corrected with calibration markers of 3 cm diameter placed on near the hip. Both measures were taken immediately before and two weeks after surgery. The value obtained by subtracting the preoperative value from the postoperative value in the surgical side was defined as the change in leg length or global offset. Postoperative leg length discrepancy which was the value obtained by subtracting the contralateral side from surgical side was evaluated. The correlations between each value and HTD before the suture at the hip intermediate and 10° extension positions were evaluated.

### Statistical analysis

A paired t-test was used to compare HTD before and after anterior capsular suture at the hip intermediate and 10° extension positions. Spearman's rank correlation coefficient was used to calculate the correlation between HTD before capsular suture and DCS, and between HTD before the suture and the change in leg length or global offset. The values were shown as mean ± standard deviation. *p* < 0.05 was considered statistically significant. All analyses were performed using GraphPad Prism version 8 (GraphPad, San Diego, CA, USA).

### Ethics statement

The study was conducted according to the tenets of the Declaration of Helsinki and was approved by the Ethics Committee and Institutional Review Board at our institute (H2020-068–2). All patients were informed about the study and informed consent was obtained from all participants.

## Results

### Comparison between HTDs before and after anterior capsular suture

**Figure 4:**
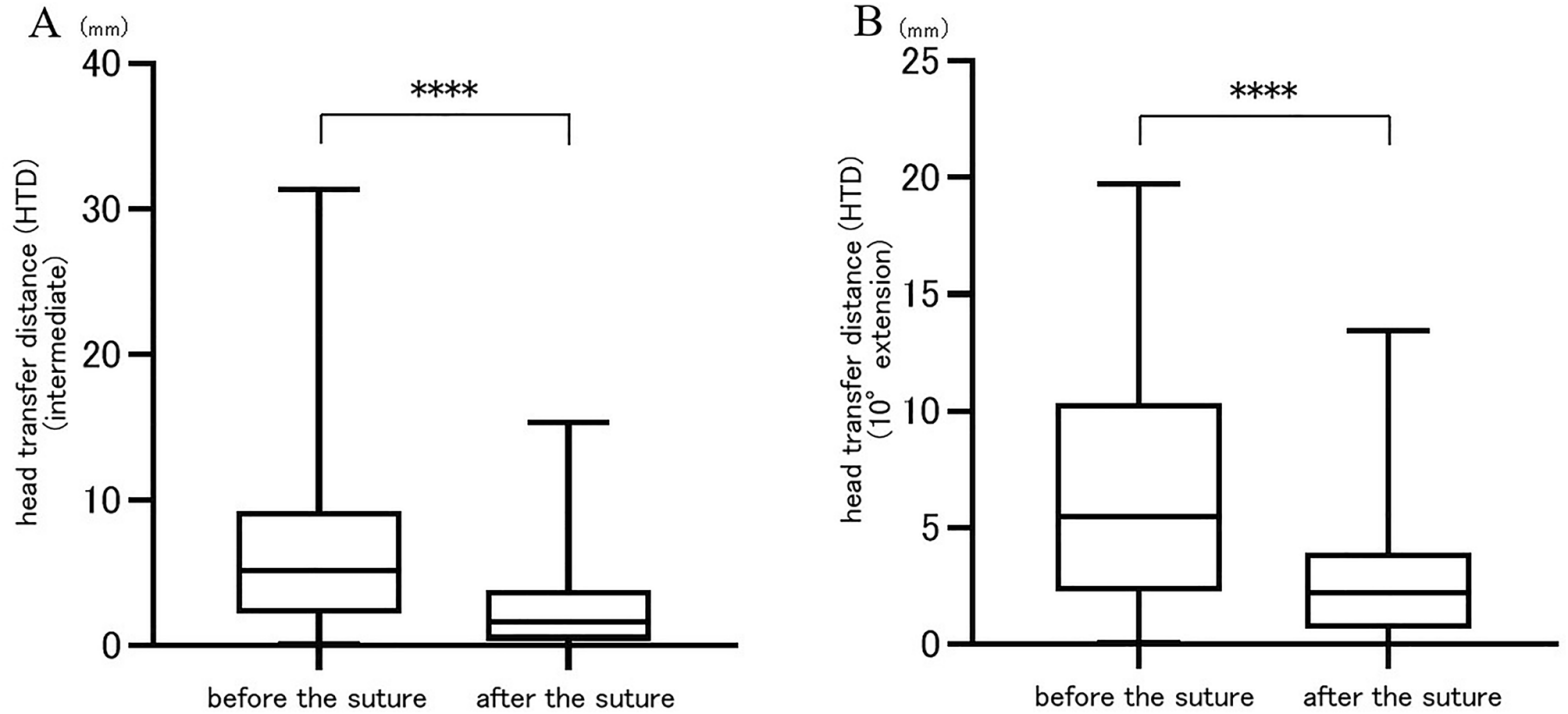
Head transfer distance (HTD) before and after anterior capsular suture in the hip intermediate (**A**) and 10°extension (**B**) positions were showed. The mean HTD after the suture were significantly decreased in both hip positions (****p < 0.0001).

**Figure 5:**
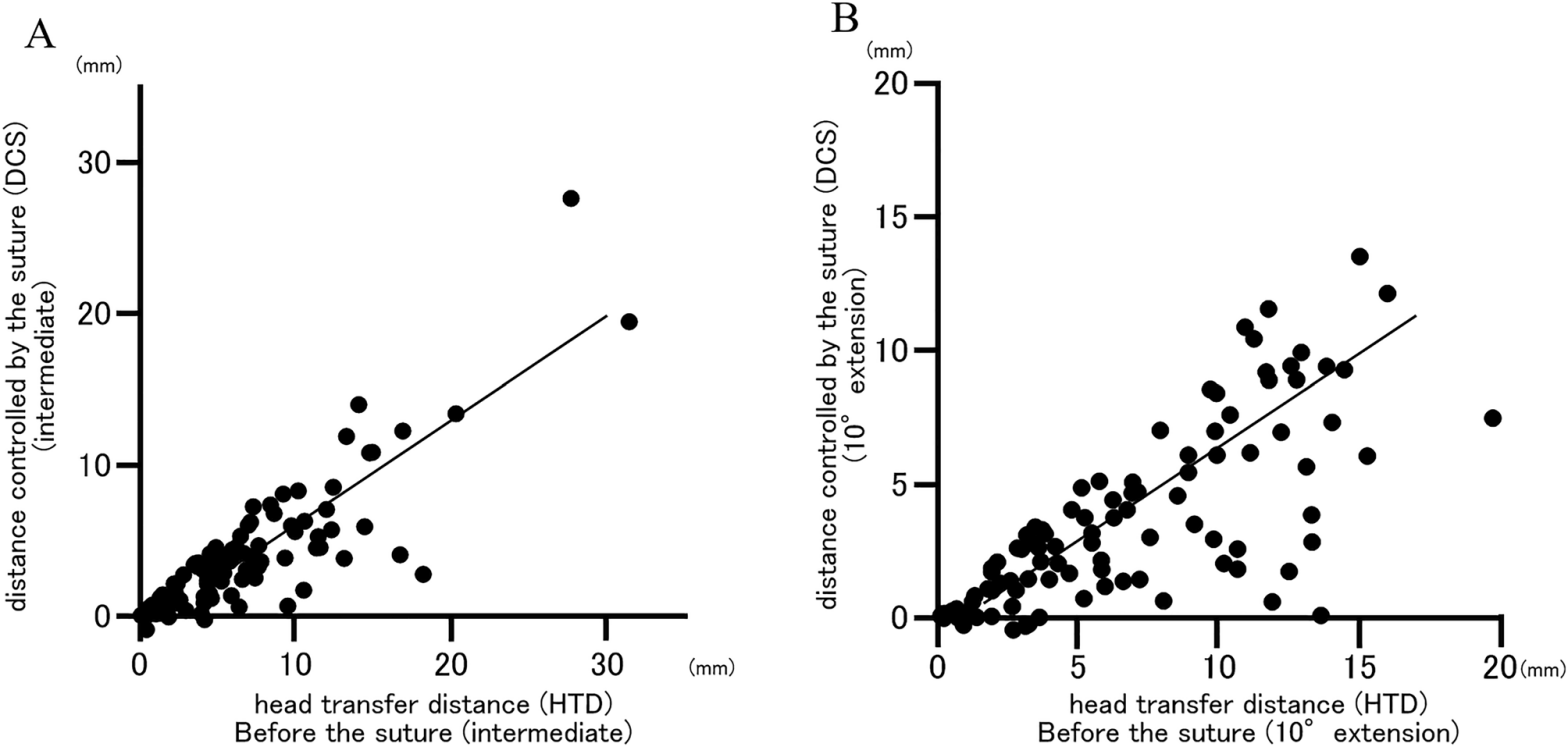
The correlations between head transfer difference (HTD) before the suture and distance controlled by the suture (DCS) in the hip intermediate (**A**) and 10°extension (**B**) positions were showed. The positive correlations were observed in both hip positions (r = 0.8623, *p* < 0.0001; r = 0.7684, *p* < 0.0001, respectively).

The mean HTD before anterior capsular suture was 5.9 ± 4.6 mm in the hip intermediate position and 6.3 ± 4.6 mm in the hip 10° extension position. HTD after the suture was 2.6 ± 2.7 mm in the hip intermediate position and 2.9 ± 3.1 mm in the 10° extension position. HTD significantly decreased after the suture in both hip positions (intermediate: *p* < 0.0001, 10°extension: *p* < 0.0001) (Fig. 4). HTD before the suture was greater in the hip extension position than intermediate position (*p* = 0.032). In contrast, there was no significant difference between HTD after the suture in the hip intermediate and extension positions (*p* = 0.078). A positive correlation was found between HTD before the suture and DCS in both the hip intermediate and 10° extension positions (r = 0.8623, *p* < 0.0001; r = 0.7684, *p* < 0.0001, respectively) (Fig. 5).

### Influence of leg length and global offset on HTD

**Figure 6:**
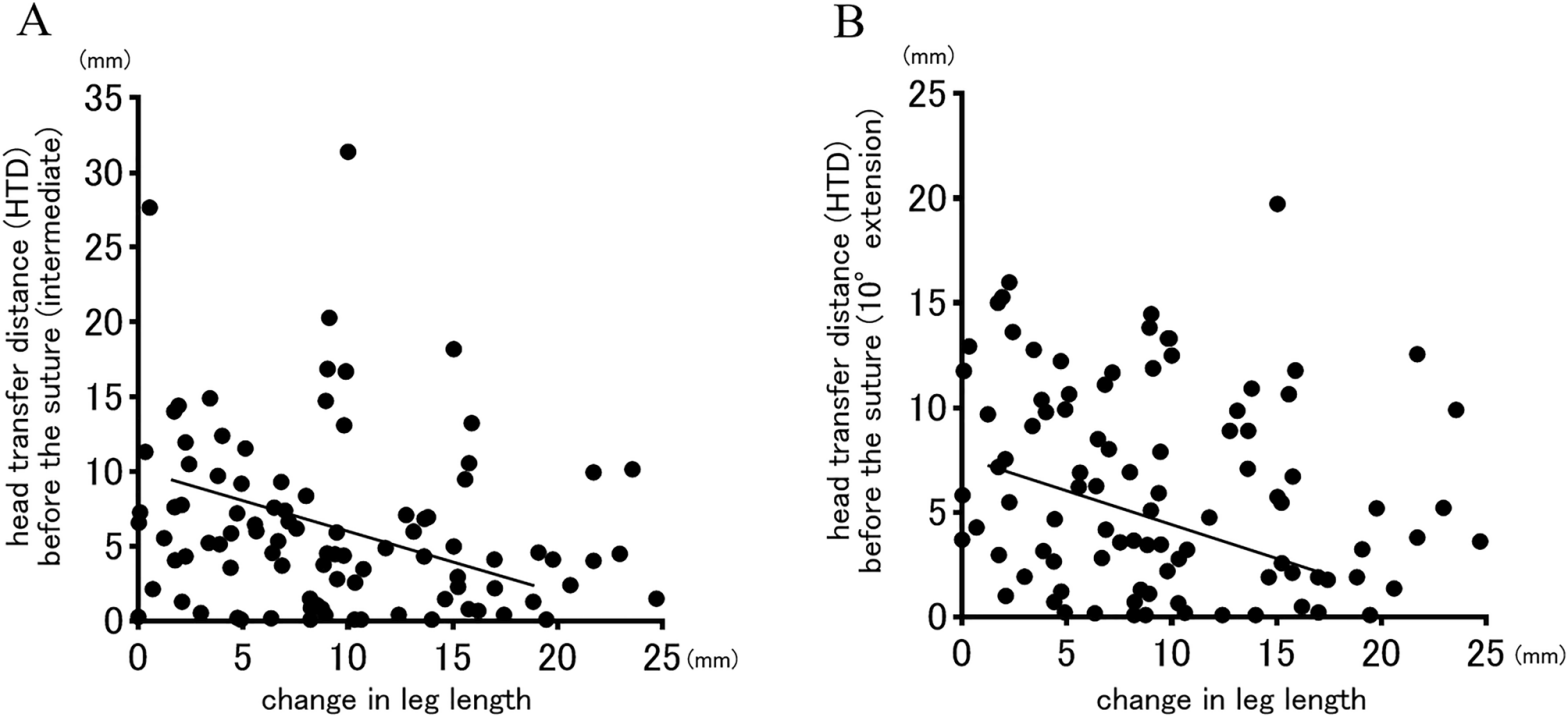
The correlations between head transfer distance (HTD) before the suture and change in leg length in the hip intermediate (**A**) and 10°extension (**B**) positions were showed. Although the negative correlations were statistically observed in both hip positions, both correlation coefficients were very low (r = -0.2124, *p* = 0.013; r = -0.2021, *p* = 0.021, respectively).

**Figure 7:**
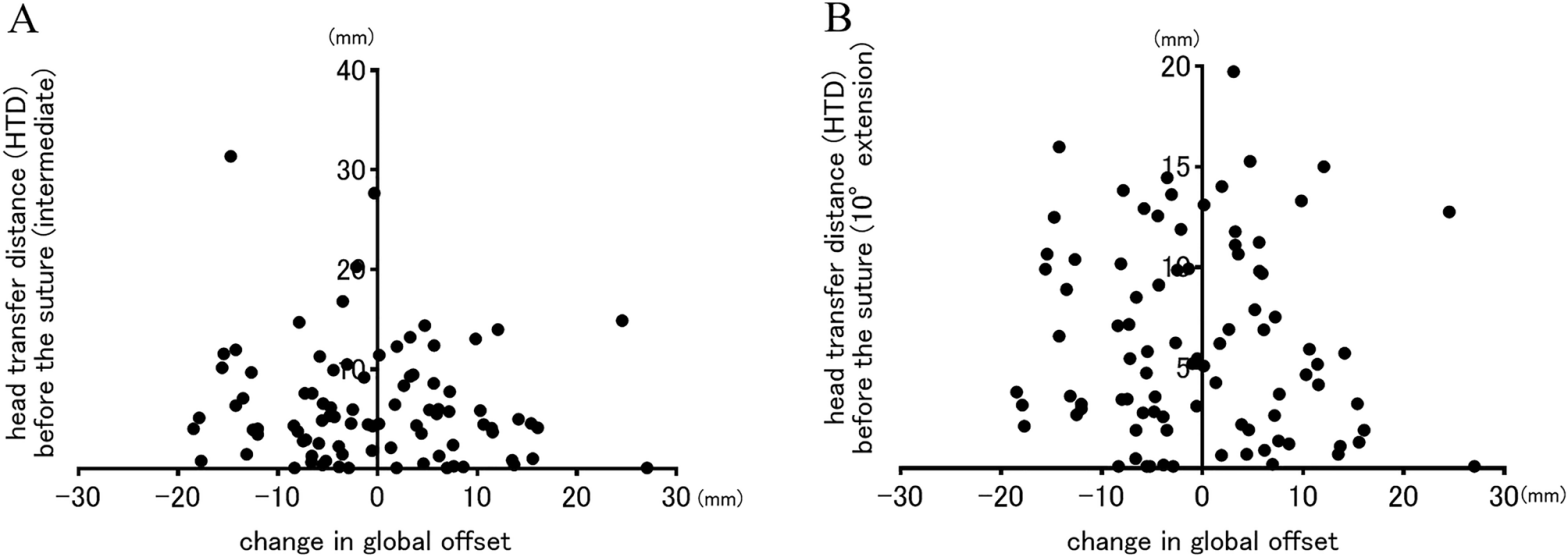
The correlations between head transfer distance (HTD) before the suture and change in global offset in the hip intermediate (**A**) and 10°extension positions (**B**) were examined. There were no significant correlations in both groups. (r = -0.068, *p* = 0.542; r = -0.082, *p* = 0.461, respectively).

In the correlation between change in leg length and HTD before the suture, although the *p* values were less than 0.05, the correlation coefficients were as low as 0.2 in both hip positions, indicating almost no correlation (intermediate: r = -0.2124, *p* = 0.013; 10°extension: r = -0.2021, *p* = 0.021) (Fig. 6). No correlations between postoperative leg length discrepancy and HTD before the suture were shown in both hip positions (intermediate: r = -0.077, *p* = 0.468; 10°extension: r = -0.077, *p* = 0.471). Regarding correlations between HTD before the suture and the change in global offset, no correlations were observed for both hip positions (intermediate: r = -0.068, *p* = 0.542, 10° extension: r = -0.082, *p* = 0.461) (Fig. 7). No patients included in this study had postoperative dislocations.

## Discussion

There are no reports on how much hip laxity changes between before and after anterior capsular suture in DAA-THA. In this study, hip laxity against axial traction force was improved after anterior capsular suture compared to before the suture in both hip intermediate and 10°extension positions. Moreover, the effect was more pronounced in cases where hip laxity before the suture was greater. This is the first report to show the contribution of anterior capsular suture to improving hip laxity in DAA-THA.

The iliofemoral ligament, including vertical and horizontal bands, contributes to the control of external and internal rotation in hip extension position, and external rotation in hip flexion position^7,8^. A cadaver study by Khair et al. reported that when anterior transverse capsulotomy was made 1 cm distal to the acetabular rim of the native hip, laxity against hip traction increased with increasing incision length^9^. Low soft tissue tension against axial traction force may not be necessarily a direct risk factor for postoperative dislocation, because axial traction force hardly causes dislocation after THA clinically. However, there is a report that hip laxity to axial traction forces may be associated with recurrent dislocation^10^. They described that the displacement of the femoral head against distal traction in patients with recurrent dislocation after THA increased fourfold compared to patients without recurrent dislocation. Thus, residual hip laxity against axial traction force would be considered a risk factor for dislocation after THA. In this study, although it is unknown if anterior capsular suture prevents dislocation, we showed that anterior capsular suture significantly improved hip laxity against axial traction force in hip intermediate and extension positions in DAA-THA. Furthermore, when Myers et al. measured the hip external rotation angle before and after iliofemoral ligament dissection in a cadaver's native hip, the external rotation angle increased after dissection^13^. External rotation is a risk position for anterior dislocation in DAA-THA and its laxity is thought to increase the risk of dislocation. From this point of view, although the effect of anterior capsular suture for laxity in external rotation has not been investigated in this study, it may contribute to preventing dislocation.

Additionally, as another clinical relevance, previous study showed that the wear of bearing surfaces was accelerated when the femoral head was separated from the insert during the swing phase of walking after THA^14^. Since in the patients with greater hip laxity against axial traction force the femoral head would be easily separated in the swing phase, the wear of bearing surface might progress more during the postoperative process. Thus, improvement of hip laxity could contribute to reduction of the risk after THA. Thus, anterior capsular suture might help improve long term clinical outcomes in DAA-THA. Further study will be needed to clarify this issue.

HTD before the suture and DCS showed a positive correlation in present study. This indicates that anterior capsular suture is the most effective to improve hip laxity in cases with greater axial transfer of the head. Conversely, some cases hardly had hip laxity before the suture, and such cases are considered less significant for the suture. Therefore, it may be also important to decide whether or not to suture anterior capsule depending on the degree of hip laxity before the suture. However, the degree of laxity that should be sutured has not been clarified in this study. We believe that this issue should be investigated in the future to make it clinically useful.

Previous studies reported that increased leg length and global offset were factors improving hip stability in THA because these reinforce soft tissue tension^15–17^. In this study, the leg lengthening or increase in global offset from preoperative value did not necessarily reduce axial transfer of the head before the suture in DAA-THA. This may depend on the degree of preoperative joint contracture in each patient. In other words, the preoperative soft tissue tension is different in each case, which may lead to differences in hip laxity before the suture even with the same amount of leg lengthening or offset change. On the other hand, leg length discrepancy is a common cause of patient complaints and litigation^18–20^. Thus, the excessive leg lengthening for improvement of hip laxity would lead to poor clinical outcomes. In such cases, the anterior capsular suture may be a useful method as it improves hip laxity without excessive leg lengthening. We suggest that an intraoperative hip laxity test through axial traction should be performed, and in cases with hip laxity, anterior capsular suture should be performed in DAA-THA.

On the other hand, concerns with capsular sutures are longer operating time, increased blood loss, and limited postoperative range of motion (ROM). A few reports showed that there were no significant differences for operating time, blood loss, and postoperative ROM compared between capsule repaired and capsule resected groups in DAA-THA^21,22^. Moreover, Stadelmann et al. also reported significantly lower blood loss in a capsule preserved group than a capsule resected group^22^. Thus, the disadvantage of anterior capsular suture may not exist in DAA-THA.

This study has several limitations. First, we did not compare capsular suture cases with resection cases. Although all patients in present study had no early postoperative dislocations, it cannot be concluded whether the dislocation rate is reduced by anterior capsular suture. In previous studies, no differences of dislocation rate were found between resection or preservation of the anterior capsule in DAA-THA^21,23^. However, these studies did not take into account the degree of hip laxity before the suture, and the cases that did not have hip laxity before the suture could be included. In present study, the cut-off value of HTD before the suture that should be sutured to prevent dislocation has not been elucidated. It is of great clinical importance to clarify this issue. Furthermore, this was a study of cases in which the cup was placed accurately using CT-based navigation system or fluoroscopy. If the cup is malalignment, the forces on the anterior capsule may be greater. In these cases, it is unclear whether the suture of the anterior capsule is strong enough to prevent early postoperative anterior dislocation, because the anterior capsule can be subjected to significant forces in the event of dislocation. To clarify these issues, a long-term, large-scale randomized controlled trial with or without anterior capsular suture is needed. Second, we did not verify whether the sutures were loose after the traction. However, there were no cases of grossly loose sutures after traction. In addition, there was no significant difference in the amount of HTD between the first and second traction. Third, we were unable to evaluate the controlled effect with respect to external rotation by the suture. External rotation stability is also important to prevent anterior dislocation in DAA-THA. Further studies should be conducted in the future. Finally, axial traction was performed to evaluate hip laxity. Although assessment of anterior laxity in the hip extension and external rotation position is essential to evaluate preventive effects of anterior dislocation in DAA-THA^12^, there are no appropriate intraoperative examinations to quantify the laxity. Thus, as previous report has mentioned the association between laxity to traction force and recurrent dislocation^10^, laxity against axial traction force was assessed in present study.

In conclusion, hip laxity against axial traction force before and after anterior capsular suture was evaluated to understand how much hip laxity changes between before and after the suture in DAA-THA. Additionally, we investigated which cases showed greater improvement in laxity against axial traction. The anterior capsular suture improved hip laxity, and the effect was particularly higher in patients with greater hip laxity before the suture. Further study will be needed to reveal how much laxity required anterior capsular suture.

## Data Availability

The datasets analyzed in this study are available from the corresponding author on reasonable request.
